# Physical Fitness Attenuates the Impact of Higher Body Mass and Adiposity on Inflammation in Women With Systemic Lupus Erythematosus

**DOI:** 10.3389/fimmu.2021.729672

**Published:** 2021-10-14

**Authors:** Sergio Sola-Rodríguez, José Antonio Vargas-Hitos, Blanca Gavilán-Carrera, Antonio Rosales-Castillo, Raquel Ríos-Fernández, José Mario Sabio, Alberto Soriano-Maldonado

**Affiliations:** ^1^ Department of Education, Faculty of Education Sciences, University of Almería, Almería, Spain; ^2^ SPORT Research Group (CTS-1024), CERNEP Research Center, University of Almería, Almería, Spain; ^3^ Systemic Autoimmune Diseases Unit, Department of Internal Medicine, “Virgen de las Nieves” University Hospital, Granada, Spain; ^4^ Department of Physical Education and Sport, Faculty of Sport Sciences, University of Granada, Granada, Spain; ^5^ Systemic Autoimmune Diseases Unit, Department of Internal Medicine, “San Cecilio” University Hospital, Granada, Spain

**Keywords:** obesity, systemic low-grade inflammation, cardiorespiratory fitness, range of motion, flexibility, autoimmune diseases, body mass index, body fat percentage (BF%)

## Abstract

**Aims:**

Higher body mass and adiposity represent independent contributors to the systemic low-grade inflammatory state often observed in patients with systemic lupus erythematosus (SLE). This study assessed the role of physical fitness in the association of body mass and adiposity with inflammation in women with SLE.

**Methods:**

A total of 77 women with SLE were included in this cross-sectional study. We obtained body mass index, waist-to-height ratio, and body fat percentage as indicators of body mass and adiposity. Inflammation was assessed through Serum levels of C-reactive protein, interleukin 6, and leptin. Cardiorespiratory fitness was assessed with the 6-minute walk test, range of motion with the back-scratch test, and muscular strength with handgrip dynamometry.

**Results:**

Cardiorespiratory fitness attenuated the association of both body mass index and body fat percentage with interleukin 6 (all, P<0.05). Range of motion attenuated the association of body mass index with interleukin 6 (P<0.05) and the association of body fat percentage with C-reactive protein (P<0.05). These interactions indicated that higher fitness was associated with a lower increase in inflammation per unit increase of body mass or adiposity. Muscular strength showed a non-significant trend to attenuate the association of body fat percentage with interleukin 6 (P=0.057) but potentiated the association of body fat percentage with leptin (P<0.05).

**Conclusion:**

These findings suggest that higher levels of cardiorespiratory fitness and range of motion might attenuate the impact of higher body mass and adiposity on inflammation in women with SLE. The role of muscular strength requires further investigation.

## 1 Introduction

Systemic lupus erythematosus (SLE) is an autoimmune disease characterized by a chronic low-grade inflammatory state that is independent of the disease activity (1). This inflammatory state, along with a higher prevalence of traditional cardiovascular risk factors, seems to contribute to the increased risk of atherosclerosis and premature cardiovascular diseases observed in this population (2, 3).

Higher body mass and adiposity represent have shown to be independent contributors to the systemic low-grade inflammatory state of patients with SLE (4, 5) that worsen the course of SLE (2) and are associated with higher disease activity and damage accrual (5). This is not surprising since adipose tissue is an active endocrine organ that secretes a variety of cytokines such as C-reactive protein (hsCRP), interleukin-6 (IL-6), and leptin, which are, in fact, involved in atherosclerosis not only in the general population (6) but also in patients with systemic autoimmune diseases (2, 7, 8). Among other relevant indicators, body mass index (BMI; a measure of body mass) (9), waist-to-height ratio (a measure of central adiposity) (10) and body fat percentage (a measure of total adiposity) (11), are independent predictors of cardiovascular diseases (2, 12).

Physical fitness is a strong modifiable health marker both in the general population (13) and in patients with rheumatological diseases (14, 15). Several studies have observed that patients with SLE present low levels of cardiorespiratory fitness (16, 17) and muscular strength (18), which are closely related to the functional status, fatigue and the quality of life of the patients (19). As low physical fitness, and particularly low cardiorespiratory fitness is a significant mortality predictor in the general population (20) that seems to be associated with higher age-related arterial stiffness (16) and a worse cardiovascular profile in patients with rheumatic conditions (14), we hypothesized that physical fitness could attenuate the impact of higher body mass and adiposity on inflammation in women with SLE. As fitness is modifiable through exercise interventions, investigating the extent to which fitness influences the association of body mass and adiposity with inflammation in patients with SLE is of clinical interest and may help to provide further insight into the potential role of exercise as an anti-inflammatory therapy in this population.

Therefore, the aim of this study was to assess the role of physical fitness in the association of body mass and adiposity with inflammation in women with SLE.

## 2 Material and Methods

### 2.1 Design and Participants

This is a cross-sectional study in which 172 patients with SLE were recruited. Women, with a diagnosis of SLE according to the ACR criteria (21), with a minimum medical follow-up of 1 year at our unit and both treatment and clinical stability (defined as no changes in the systemic lupus erythematosus disease activity index [SLEDAI]) during the previous 6 months of the study were included. Exclusion criteria were not being able to read, understand and/or sign the informed consent; personal history of clinical cardiovascular diseases in the previous year, receiving a biological treatment or requiring doses of prednisone (or equivalent) greater than 10 mg/day during the previous 6 months of the study. Detailed information about the aims and study procedures was given to all the participants, who signed informed consent before being included in the study. The Research Ethics Committee reviewed and approved the study protocol.

### 2.2 Sample Size Calculation

The sample size was calculated for a clinical trial (NCT03107442) about the effects of aerobic exercise on arterial stiffness (primary outcome), inflammation, fitness (secondary outcomes) and patient-reported outcomes that was published earlier (22, 23). A total of 58 women with SLE were recruited for the trial, although a larger sample (n=77) performed baseline evaluations for cross-sectional analyses. Therefore, the nature of this study can be considered exploratory.

### 2.3 Procedures

Potentially eligible participants were invited by phone to a personal screening. Included participants attended the Hospital facilities on two different occasions. On day 1, socio-demographic and clinical information were collected, and anthropometric measures and physical fitness tests performed. On day 2 (i.e. between 2 and 4 days after day 1), 8-h fasting blood samples were collected between 8:00 am and 10:00 am.

### 2.4 Outcome Measures

#### 2.4.1 Body Mass and Adiposity Assessment

Height (cm) was measured using a height gauge, weight (kg) and body fat percentage with a bioimpedance device (InBody R20, Biospace, Seoul, Korea), and BMI was calculated (kg/m^2^). Waist circumference (cm) was measured with an anthropometric tape (Harpenden, Holtain Ltd., Wales, UK), and waist-to-height ratio (waist circumference/height) was calculated.

#### 2.4.2 Blood Samples and Biochemical Analyses

Fasting blood specimens for biochemical and immunological tests were collected and routinely processed by the central laboratory of our hospital.

Regarding inflammatory markers, high-sensitivity CRP and interleukin 6 (IL-6) were measured in serum, which was initially separated by centrifugation and stored at –70°C. CRP levels were assessed by an immunoturbidimetric method using the ARCHITECT cSystems; MULTIGENT CRP Vario assay. Bioserum concentration of IL-6 (pg/mL) was measured by immunoradiometric assay using commercial kits (MILLIPLEX MAP Kit Human High Sensitivity T Cell Magnetic Bead Panel [HSTMAG-28SK], Millipore) following the manufacturer’s instructions. Quantitative data were obtained by using the Luminex-200 system (Luminex Corporation, Austin, TX), and data analysis was performed on XPonent 3.1 software. The detections limits for IL-6 were 0.73 pg/mL.

Leptin was measured in serum by the enzyme-linked immunosorbent assay DBC Direct Kit (Diagnostic Biochem. Canada, Canada) with 0.5 ng/ml sensitivity. Intra assay coefficient of variation (CV) and interassay CV of the kit were 5.9 and 3.7%, respectively.

#### 2.4.3 Physical Fitness

Cardiorespiratory fitness was assessed through the 6-minute walk test. The 6-minute walk test measures the maximum distance (in meters) a person is able to walk during six minutes (24). Previously, this test has been commonly used to investigate cardiorespiratory fitness in rheumatic diseases, including patients with SLE (25–27).

Upper-body range of motion, also referred to as flexibility, was assessed with the back-scratch test (24). This test measures how close the hands can be brought together behind the back. The distance between (or overlap of) the middle fingers behind the back was recorded twice for each arm, and the best scores from the right and left arms were averaged.

Muscular strength was assessed with the handgrip strength test as previously described (28). In this test, the subject holds a dynamometer in the hand, with the arm at right angles and the elbow by the side of the body. When ready, the subject squeezes the dynamometer with maximum isometric effort for about 5 seconds. The best result after two trials for each hand is recorded, with at least 30 seconds recovery between each effort, and best score of each hand was used to compute an average of the two hands.

#### 2.4.4 Other Measurements

All participants filled out a socio-demographic and clinical data questionnaire, information that was completed consulting a computerized database of the patients that included age, educational level, occupational status, and SLE data (diagnostic criteria, year of diagnosis, time of evolution, and treatments). Disease activity was assessed through SLEDAI, which takes into account the presence/absence of several clinical and analytical manifestations; the final score ranges from 0 to 105, where a higher score indicates higher degree of disease activity (29). Blood pressure was measured with Mobil-O-Graph^®^ (IEM GmbH, Stolberg, Germany) (30).

### 2.5 Statistical Analysis

The descriptive characteristics of the study participants are presented as median and interquartile range for continuous variables, and frequencies and percentages for categorical variables, unless otherwise indicated. Two inflammatory markers (i.e. IL-6 and hsCRP) were winsorized due to the presence of 1 and 3 outliers, respectively. The distribution of the main study variables assessed through histograms, Kolmogorov-Smirnov Test, and Q–Q charts, showed a non-normal distribution. Consequently, non-parametric tests were used. Quantile regression models were built, including each inflammatory marker as dependent variable in separate models and the body mass or adiposity indicator, fitness, and the body mass/adiposity×fitness interaction as independent variables. Age, SLEDAI, and corticosteroid intake were entered as potential confounders (31). Whenever the interaction was not significant, it was removed from the models and the results are presented without the interaction term.

The statistical analyses were performed with Stata v.14.0 (Stata Corp LP., Texas, USA. Statistical significance was set at P<0.05.

## 3 Results

**Figure 1:**
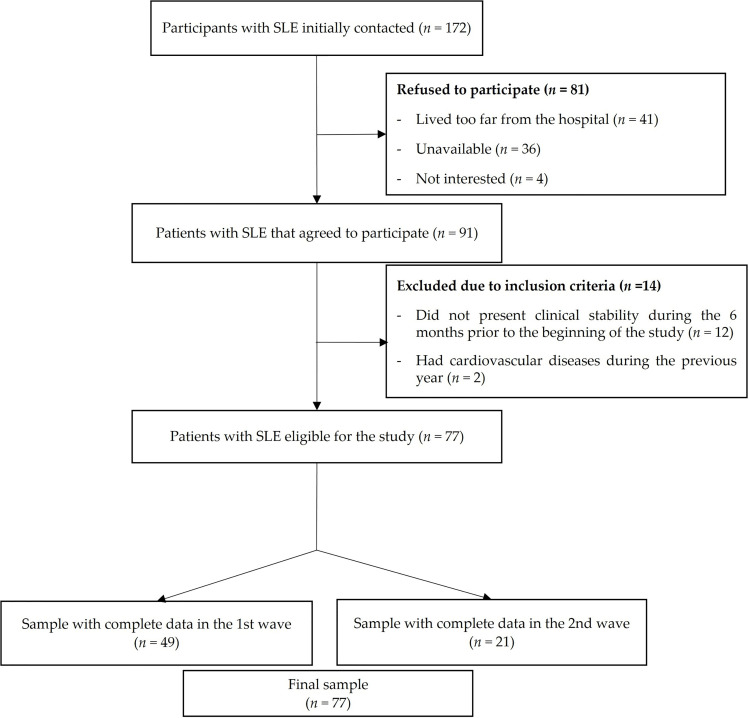
Flow diagram of the study participants throughout the study.

The flowchart of the study participants is presented in **Figure 1**. From a total of 172 patients initially invited, 81 refused to participate (41 patients reported living very far from the hospital, 36 were not able to find time to perform the evaluations, and 4 were not interested), 12 patients did not present clinical stability during the previous 6 months at the beginning of the study, and 2 patients had cardiovascular disease during the previous year. A total of 77 women with SLE (mean age 43.2, SD 13.8) fulfilled the inclusion criteria, agreed to participate, and were assessed in two waves (49 women in October 2016 and 28 women in February 2017). Both evaluations were identical, with the exception that 6-minute walk test, IL-6 and CRP were not carried out (n=28) in 2017 wave due to timing issues. One woman did not perform both handgrip strength test and back-scratch test due to a wrist injury.

The descriptive characteristics of the study participants are presented in **Table 1**. The median BMI was 24.0 (IQR 22.5 – 27.3) kg/m^2^, the median waist-to-height ratio was 0.48 (IQR 0.47 – 0.55) cm and the median body fat percentage was 34.5 (IQR 28.9 – 40.5). Regarding inflammatory variables, the median hsCRP levels were 1.77 mg/L (IQR 0.7 – 3.12), the median IL-6 levels were 2.16 (IQR 1 – 4.23) pg/mL, and the median leptin levels were 28.8 ng/mL (IQR 19.15 – 52.55). For cardiorespiratory fitness, the median walking distance assessed with the 6-minute walk test was 575 (IQR 525 – 625) meters. For muscular strength, the median handgrip strength was 24.2 (IQR 20.2 – 26.5) kg. For range of motion, the average score in the back-scratch test was 0.1 (IQR -7.5 – 7.25) cm.

**Table 1:** Descriptive characteristics of the study participants.

	N	Median	IQR
Age (years)	77	42.7	32.6 – 53.88
Weight (kg)	77	62.7	57.9 – 69.0
Height (cm)	77	159.5	155 – 164
Body Mass Index (kg/m^2^)	77	24.0	22.5 – 27.3
Waist-to-Height Ratio (cm)	77	0.48	0.465 – 0.552
Body Fat (%)	77	34.5	28.9 – 40.5
Waist circumference (cm)	77	78.0	73.4 – 87.0
6-Minute Walk Test (meters)	49	575	525 – 625
Back-scratch Test (cm)	76	0.125	-7.5 – 7.25
Dominant back-scratch Test (cm)	76	3.0	-4.5 – 8.25
Non dominant back-scratch Test (cm)	76	-2.75	-11.5 – 6.0
Handgrip Strength (kg)	76	24.2	20.2 – 26.5
Dominant Handgrip Strength (kg)	76	24.05	20.3 – 27.25
Non dominant Handgrip Strength (kg)	75	24.0	20.3 – 27.0
Interleukin 6 (pg/mL)	44	2.16	1.0 – 4.23
hsCRP (mg/L)	77	1.77	0.7 – 3.12
Leptin (ng/mL)	44	28.75	19.15 – 52.55
Dyslipidemia (n, %)	77	14 (18)	
Diabetes (n, %)	77	1 (1)	
Smokers (n, %)	77	45 (58)	
Duration of SLE (years)	77	12	6 – 21
SLEDAI^*^	77	0.68	1.5
SDI^*^	77	0.55	1.11
Cumulative Prednisone dose (mg)	77	2547.5	0 – 5056.25
Daily Prednisone dose (mg)	77	2.5	0 – 5
	**n (%)**		
Prednisone use (%)	77	50 (65)	
Immunosuppressants (%)	77	35 (45)	
Antimalarials (%)	77	69 (89)	
NSAIDs intake (%)	77	0 (0)	

IQR, Interquartile range; hsCRP, High sensitivity C-reactive protein; SLEDAI, Systemic lupus erythematosus disease activity index; NSAIDs, Nonsteroidal anti-inflammatory drugs; SLE, Systemic lupus erythematosus. SDI, Systemic Lupus International Collaborating Clinics/American College of Rheumatology Damage Index. All variables show mean and SD values except corticosteroid dose, immunosuppressants, dyslipidemia, diabetes, and smokers. ^*^Mean and standard deviation.

**Tables 2**–**4** represent the association of body mass/adiposity and physical fitness (and their interaction) with inflammation after controlling for potential confounders. Regarding cardiorespiratory fitness, there was a significant interaction with BMI on IL-6 (P<0.05), indicating that higher cardiorespiratory fitness was associated with a lower increase in IL-6 per each additional BMI unit. For example, participants who were able to walk 380 meters in the 6-minute walk test presented an increase of 1.13 pg/mL in IL-6 for 1 incremental BMI unit, while those who were able to walk 560 meters presented an increase of 0.12 pg/mL in IL-6 for 1 incremental BMI unit. There was also a significant interaction of cardiorespiratory fitness with body fat percentage on IL-6 (P<0.05). Range of motion also interacted with BMI on IL-6 (P<0.05) so that, for example, a score of -20 cm in the back-scratch test was associated with an increase of 0.80 pg/mL in IL-6 for 1 incremental BMI unit, while a score of +1 cm was associated with an increase of 0.07 pg/mL in IL-6 for 1 incremental BMI unit. Furthermore, there was an interaction with body fat percentage on hsCRP (P<0.05). Finally, there was an interaction of muscular strength with body fat percentage on leptin (P<0.05). There were also non-significant interactions, such as interaction of range of motion with BMI on hsCRP (P=0.056), the interaction of cardiorespiratory fitness with waist-to-height ratio on IL-6 (P=0.078), and the interaction of muscular strength with body fat percentage on IL-6 (P=0.057). A graphical representation of the main study findings presenting the key interactions of body mass/adiposity with physical fitness on inflammatory markers is displayed in **Figure 2**.

**Table 2:** Quantile regression analyses assessing the interaction of body mass/adiposity with cardiorespiratory fitness on inflammatory markers.

hsCRP						
	B	SE	95% CI			p
BMI	0.209	0.063	0.082	,	0.336	**0.002**
CRF	0.01	0.005	-0.001	,	0.02	0.065
BMI×CRF						NS*
WHtR	4.661	5.193	-5.811	,	15.133	0.374
CRF	0.005	0.005	-0.006	,	0.016	0.371
WHtR×CRF						NS*
BF%	0.048	0.038	-0.028	,	0.124	0.213
CRF	0.006	0.005	-0.048	,	0.017	0.268
BF%×CRF						NS*
**IL-6**						
	**B**	**SE**	**95% CI**			**p**
BMI	5.504	1.478	2.509	,	8.5	**0.001**
CRF	0.229	0.071	0.085	,	0.372	**0.003**
BMI×CRF	-0.009	0.003	-0.015	,	-0.004	**0.002**
WHtR	142.8	71.72	-2.522	,	288.1	0.054
CRF	0.122	0.069	-0.019	,	0.263	0.088
WHtR×CRF	-0.245	0.135	-0.519	,	0.029	0.078
BF%	1.351	0.537	0.264	,	2.438	**0.016**
CRF	0.076	0.035	0.004	,	0.148	**0.039**
BF%×CRF	-0.002	0.001	-0.004	,	0	**0.026**
**Leptin**						
	**B**	**SE**	**95% CI**			**p**
BMI	3.188	0.568	2.038	,	4.338	**<0.001**
CRF	0.016	0.047	-0.079	,	0.111	0.741
BMI×CRF	NS*					
WHtR	-356.93	295.76	-956.21	,	242.33	0.235
CRF	-0.587	0.283	-1.16	,	-0.013	**0.045**
WHtR×CRF	NS*					
BF%	2.442	0.346	1.742	,	3.142	**<0.001**
CRF	0	0.048	-0.097	,	0.097	0.997
BF%×CRF						NS*

The markers of inflammation were high sensitivity C-reactive protein (hsCRP), interleukin 6 (IL-6) and leptin. The markers of body mass/adiposity were body mass index (BMI), waist-to-height ratio (WHtR) and body fat percentage (BF%); CRF, cardiorespiratory fitness.

B, unstandardized regression coefficient indicating the expected unit change in the dependent variable for one-unit change in the independent variable; SE, standard error NS, non-significant.

Quantile regression models were built including each inflammatory marker as dependent variable in separate models and the body mass or adiposity indicator, CRF, and the body mass/adiposity×CRF interaction as independent variables. All the analyses were adjusted for age, SLEDAI, and accumulated corticosteroid intake. When the interaction was not significant, the interaction term was removed from the regression model and the results are presented without interaction (i.e., the independent association of body mass/adiposity and CRF with the inflammatory marker).

**Table 3:** Quantile regression analyses assessing the interaction of body mass/adiposity with range of motion on inflammatory markers.

hsCRP						
	B	SE	95% CI			p
BMI	-0.014	0.067	-0.148	,	0.12	0.835
ROM	0.203	0.135	-0.066	,	0.473	0.137
BMI×ROM	-0.01	0.005	-0.021	,	0.0003	0.056
WHtR	3.359	4.472	-5.561	,	12.281	0.455
ROM	-0.054	0.034	-0.123	,	0.139	0.116
WHtR×ROM						NS*
BF%	-0.02	0.03	-0.08	,	0.04	0.496
ROM	0.222	0.087	0.048	,	0.397	**0.013**
BF%×ROM	-0.008	0.002	-0.013	,	-0.003	**0.001**
**IL-6**						
	**B**	**SE**	**95% CI**			**p**
BMI	0.109	0.203	-0.302	,	0.52	0.594
ROM	0.932	0.443	0.035	,	1.829	**0.042**
BMI×ROM	-0.035	0.017	-0.068	,	-0.001	**0.045**
WHtR	1.845	10.924	-20.27	,	23.96	0.867
ROM	0.06	0.074	-0.089	,	0.21	0.417
WHtR ×ROM						NS*
BF%	0.135	0.08	-0.028	,	0.298	0.102
ROM	0.07	0.073	-0.078	,	0.218	0.34
BF%×ROM						NS*
**Leptin**						
	**B**	**SE**	**95% CI**			**p**
BMI	3.636	0.558	2.507	,	4.766	<0.001
ROM	0.433	0.323	-0.221	,	1.086	0.188
BMI×ROM						NS*
WHtR	253.53	52.62	147.01	,	360.06	**<0.001**
ROM	0.17	0.4	-0.639	,	0.98	0.673
WHtR×ROM						NS*
BF%	2.374	0.402	1.56	,	3.189	**<0.001**
ROM	-0.064	0.392	-0.859	,	0.73	0.871
BF%×ROM						NS*

The markers of inflammation were high sensitivity C-reactive protein (hsCRP), interleukin 6 (IL-6) and leptin. The markers of body mass/adiposity were body mass index (BMI), waist-to-height ratio (WHtR) and body fat percentage (BF%); ROM, range of motion (ROM).

B, unstandardized regression coefficient indicating the expected unit change in the dependent variable for one-unit change in the independent variable; SE, standard error; NS, non-significant.

Quantile regression models were built including each inflammatory marker as dependent variable in separate models and the body mass or adiposity indicator, ROM, and the body mass/adiposity×ROM interaction as independent variables. All the analyses were adjusted for age, SLEDAI, and accumulated corticosteroid intake. When the interaction was not significant, the interaction term was removed from the regression model and the results are presented without interaction (i.e., the independent association of body mass/adiposity and ROM with the inflammatory marker).

**Table 4:** Quantile regression analyses assessing the interaction of body mass/adiposity with muscular strength on inflammatory markers.

hsCRP						
	B	SE	95% CI			p
BMI	0.181	0.056	0.069	,	0.293	**0.002**
HGS	-0.05	0.052	-0.154	,	0.054	0.342
BMI×HGS						NS*
WHtR	5.574	4.352	-3.109	,	14.258	0.205
HGS	-0.038	0.062	-0.162	,	0.085	0.539
WHtR×HGS						NS*
BF%	0.054	0.033	-0.011	,	0.121	0.103
HGS	-0.026	0.057	-0.141	,	0.089	0.653
BF%×HGS						NS*
**IL-6**						
	**B**	**SE**	**95% CI**			**p**
BMI	0.067	0.187	-0.312	,	0.446	0.723
HGS	-0.013	0.163	-0.342	,	0.317	0.939
BMI×HGS						NS*
WHtR	0.407	9.671	-19.17	,	19.98	0.967
HGS	0.002	0.114	-0.228	,	0.233	0.983
WHtR ×HGS						NS*
BF%	0.571	0.276	0.012	,	1.129	**0.046**
HGS	0.751	0.429	-0.118	,	0.354	0.989
BF%×HGS	-0.021	0.107	-0.043	,	0	0.057
**Leptin**						
	**B**	**SE**	**95% CI**			**p**
BMI	3.193	0.526	2.128	,	4.259	**<0.001**
HGS	-0.282	0.564	-1.424	,	0.859	0.619
BMI×HGS						NS*
WHtR	264.53	35.574	192.52	,	336.55	**<0.001**
HGS	0.543	0.5	-0.47	,	1.557	0.285
WHtR×HGS						NS*
BF%	-0.231	1.094	-2.448	,	1.985	0.834
HGS	-3.095	1.735	-6.611	,	0.42	0.083
BF%×HGS	0.106	0.043	0.019	,	0.194	**0.018**

The markers of inflammation were high sensitivity C-reactive protein (hsCRP), interleukin 6 (IL-6) and leptin. The markers of body mass/adiposity were body mass index (BMI), waist-to-height ratio (WHtR) and body fat percentage (BF%); HGS, handgrip strength (HGS).

B, unstandardized regression coefficient indicating the expected unit change in the dependent variable for one-unit change in the independent variable; SE, standard error NS, non-significant.

Quantile regression models were built including each inflammatory marker as dependent variable in separate models and the body mass or adiposity indicator, HGS, and the body mass/adiposity×HGS interaction as independent variables. All the analyses were adjusted for age, SLEDAI, and accumulated corticosteroid intake. When the interaction was not significant, the interaction term was removed from the regression model and the results are presented without interaction (i.e., the independent association of body mass/adiposity and HGS with the inflammatory marker).

**Figure 2:**
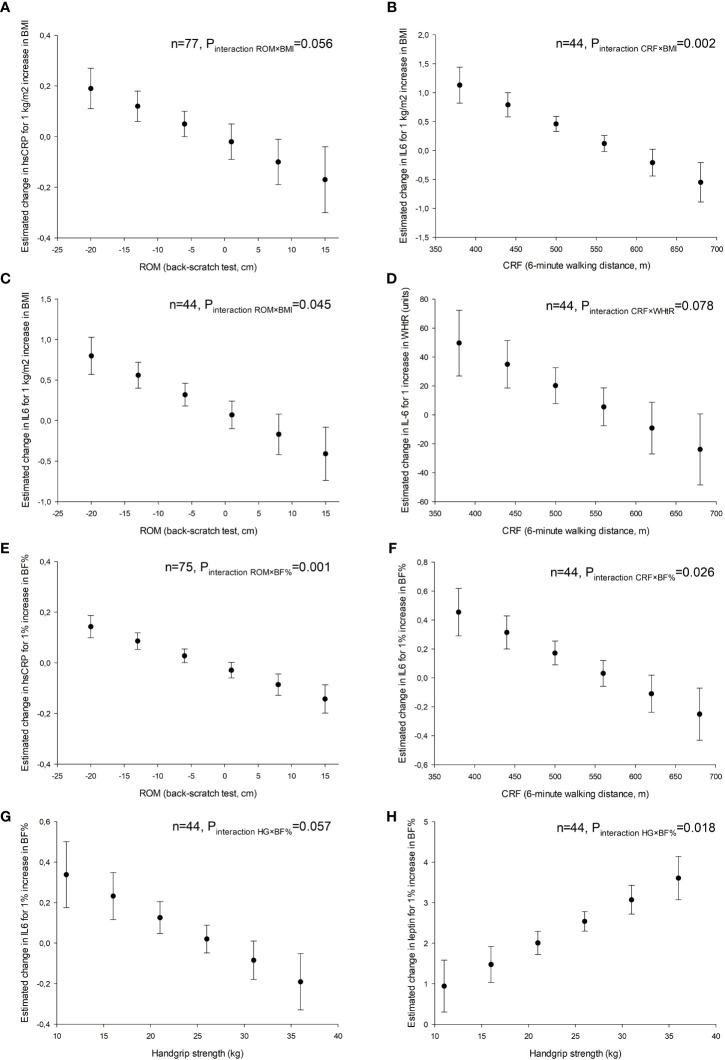
Graphical representation of the interaction of body mass and adiposity with physical fitness on inflammation in women with systemic lupus erythematosus. **(A, C, E)** ROM, upper-body range of motion assessed by the back-scratch test. **(B, D, F)** CRF, cardiorespiratory fitness assessed by the distance walked in the 6-minute walk test. **(G, H)** Muscular strength assessed with handgrip dynamometry. BMI, body mass index; BF%, body fat percentage; WHtR, waist-to-height ratio; hsCRP, high sensitivity C-reactive protein; IL6, interleukin6.

## 4 Discussion

The main findings of this study suggest that higher levels of physical fitness might attenuate the impact of higher body mass and adiposity on inflammation in women with SLE. Overall, we observed that higher fitness was associated with lower increase of some inflammatory markers for each additional unit of body mass/adiposity. These findings open a window of opportunity to understand the potential of fitness to counteract the effect of higher body mass and adiposity on inflammation in autoimmune diseases, although this needs to be corroborated in future and larger prospective studies.

To the best of our knowledge, this is the first study evaluating how the association of body mass and adiposity with relevant inflammatory markers might be dependent of physical fitness in women with SLE. Although the association of body mass and adiposity with inflammation is well-described both in the general population (32–34) and in SLE (4), research analyzing the potential role of fitness in this association is limited and inconclusive. While Park etal. (35) found an interaction effect of cardiorespiratory fitness with waist circumference on IL-6 in young adults, Bergens, Nilsson and Kadi (36) did not found an interaction effect when considering cardiorespiratory fitness and adiposity with pro and anti-inflammatory biomarkers in a sample of older women.

In this study, higher cardiorespiratory fitness was associated with lower increase in IL-6 per additional unit of either BMI, body fat percentage and, to a lesser extent, of waist-to-height ratio. These findings suggest that the association of body mass and adiposity with inflammation in SLE could depend on the level of cardiorespiratory fitness and that SLE patients with lower cardiorespiratory fitness levels could have higher risk of obesity-related low-grade inflammation. Cardiorespiratory fitness is a relevant health-related parameter that strongly predicts mortality risk in the general population (13) and is associated with a more favorable body composition (37) and higher health-related quality of life in SLE (38). These results extend the potential beneficial roles of cardiorespiratory fitness in women with SLE.

We also observed that higher levels of upper-body range of motion were associated with lower increase in IL-6 per unit of both BMI, and with lower increase in hsCRP per additional unit of body fat percentage. Evidence regarding the role of range of motion on health in patients with SLE is scarce, although its potential has recently gained attention in other populations (39–41). For instance, greater range of motion has been related with lower cardiometabolic risk (42) and a more favorable cardiovascular profile (43) in perimenopausal women, and with lower risk of metabolic syndrome in older adults (44). Further research on the association of range of motion with health-related parameters in autoimmune diseases is needed. Taken together, these findings suggest that the low-grade inflammatory profile associated with higher body mass and adiposity in SLE could be attenuated in people with higher range of motion, which also needs to be confirmed or contrasted.

Our results corroborated prior research underlining that higher body mass and adiposity is associated with higher leptin concentrations (45, 46). However, we failed to observe that fitness attenuated the association of body mass and adiposity with leptin. In fact, higher muscular strength was surprisingly related to higher increase in leptin per additional unit of body fat percentage. This particular result is difficult to explain and further research is needed to understand the rationale behind it. In fact, previous research showed that resistance training decreases plasma leptin levels in elderly women (47), which to some extent contrasts this observation.

Physical fitness has previously shown to attenuate the detrimental effect that obesity has on cardiovascular mortality in the general population (48). We might speculate that one of the mechanisms by which fitness attenuates this association is through attenuating the impact of obesity on inflammation. Obesity is present in almost 50% of women with SLE (8) and adipose tissue has the capacity not only to recruit and activate mononuclear cells (49) but also to produce key inflammatory cytokines, such as IL-6, which stimulates the production of CRP and other acute phase proteins by the liver (50). Therefore, the potential role of fitness in this association is of research and clinical relevance and requires further investigation, particularly in autoimmune diseases, because all the components of fitness can be enhanced through exercise programs.

This study has limitations. The cross-sectional design precludes establishment of causal relationships; therefore, we do not know whether increasing fitness through exercise programs will have an impact on the obesity-inflammation relationship. The sample size was relatively small, particularly for the leptin and IL-6 analyses, and thus they need to be confirmed or contrasted in future prospective and experimental research with larger sample sizes. Finally, the study was performed only in women with SLE with low or inactive disease; thus, we do not know whether these results apply to men or to women with higher disease activity.

In conclusion, the findings of the present study suggest that higher levels of physical fitness, particularly cardiorespiratory fitness and range of motion, might attenuate the association of higher body mass and adiposity with inflammation in women with SLE. These results underline a potential mechanism by which fitness might mitigate the effect of obesity on cardiovascular disease, although they must be corroborated in future prospective and experimental research.

## Data Availability Statement

The original contributions presented in the study are included in the article/supplementary material. Further inquiries can be directed to the corresponding author.

## Ethics Statement

The studies involving human participants were reviewed and approved by the Research Ethics Committee of Granada. The participants provided written informed consent to participate in this study.

## Author Contributions

Conceptualization, SS-R, JV-H, and AS-M. Data curation, JVH and AS-M. Formal analysis, SS-R, BG-C, and AS-M. Funding acquisition, JVH, JS, and AS-M. Investigation, SS-R, JVH, BG-C, AR-C, RR-F, JM, and AS-M. Methodology, BG-C, JV-H, and AS-M. Project administration, JV-H. Resources, AR-C, RR-F, JM, and AS-M. Supervision JV-H and AS-M. Visualization, AR-C. Writing—Original draft, SS-R, JVH, and AS-M. Writing—Review & editing, SS-R, JV-H, BG-C, AR-C, RR-F, JS, and AS-M. All authors contributed to the article and approved the submitted version.

## Funding

This work was funded by the Consejería de Salud, Junta de Andalucía (grant numbers: PI-0525-2016 and PIER-0223-2019). BG-C was supported by the Spanish Ministry of Education (FPU15/00002). AS-M was supported by the Spanish Ministry of Science, Innovation and Universities (ref. RTI2018–093302-A-I00). The funders had no role in the study design, data collection and analysis, decision to publish, or preparation of the manuscript.

## Conflict of Interest

The authors declare that the research was conducted in the absence of any commercial or financial relationships that could be construed as a potential conflict of interest.

## Publisher’s Note

All claims expressed in this article are solely those of the authors and do not necessarily represent those of their affiliated organizations, or those of the publisher, the editors and the reviewers. Any product that may be evaluated in this article, or claim that may be made by its manufacturer, is not guaranteed or endorsed by the publisher.
